# Human Ocular Epithelial Cells Endogenously Expressing SOX2 and OCT4 Yield High Efficiency of Pluripotency Reprogramming

**DOI:** 10.1371/journal.pone.0131288

**Published:** 2015-07-01

**Authors:** Ming-Wai Poon, Jia He, Xiaowei Fang, Zhao Zhang, Weixin Wang, Junwen Wang, Fangfang Qiu, Hung-Fat Tse, Wei Li, Zuguo Liu, Qizhou Lian

**Affiliations:** 1 Department of Medicine, The University of Hong Kong, Hong Kong SAR, China; 2 The HKU Shenzhen Institute of Research and Innovation, The University of Hong Kong, Hong Kong SAR, China; 3 Department of Ophthalmology, Li Ka Shing Faculty of Medicine, The University of Hong Kong, Hong Kong SAR, China; 4 Eye Institute of Xiamen University, Fujian Provincial Key Laboratory of Ophthalmology and Visual Science, Xiamen, Fujian, 361005, China; 5 Department of Biochemistry, The University of Hong Kong, Hong Kong SAR, China; University of Pécs Medical School, HUNGARY

## Abstract

A variety of pluripotency reprogramming frequencies from different somatic cells has been observed, indicating cell origin is a critical contributor for efficiency of pluripotency reprogramming. Identifying the cell sources for efficient induced pluripotent stem cells (iPSCs) generation, and defining its advantages or disadvantages on reprogramming, is therefore important. Human ocular tissue-derived conjunctival epithelial cells (OECs) exhibited endogenous expression of reprogramming factors OCT4A (the specific OCT 4 isoform on pluripotency reprogramming) and SOX2. We therefore determined whether OECs could be used for high efficiency of iPSCs generation. We compared the endogenous expression levels of four pluripotency factors and the pluripotency reprograming efficiency of human OECs with that of ocular stromal cells (OSCs). Real-time PCR, microarray analysis, Western blotting and immunostaining assays were employed to compare OECiPSCs with OSCiPSCs on molecular bases of reprogramming efficiency and preferred lineage-differentiation potential. Using the traditional KMOS (*KLF4*, *C-MYC*, *OCT4* and *SOX2*) reprogramming protocol, we confirmed that OECs, endogenously expressing reprogramming factors OCT4A and SOX2, yield very high efficiency of iPSCs generation (~1.5%). Furthermore, higher efficiency of retinal pigmented epithelial differentiation (RPE cells) was observed in OECiPSCs compared to OSCiPSCs or skin fibroblast iMR90iPSCs. The findings in this study suggest that conjunctival-derived epithelial (OECs) cells can be easier converted to iPSCs than conjunctival-derived stromal cells (OSCs). This cell type may also have advantages in retinal pigmented epithelial differentiation.

## Introduction

Introduction of exogenous reprogramming factors *KLF4*, *C-MYC*, *OCT4* and *SOX2* (KMOS) reprograms somatic cells to induced pluripotent stem cells (iPSCs) [1, 2]. Recent developments in reprogramming techniques using episome or mRNA-based assay have resulted in the successful generation of iPSCs without integration of exogenous components or genes in genome. These techniques in turn facilitate the applications of iPSCs in personalized regenerative and pharmaceutical medicine[3]. Nonetheless there remain many challenges remain prior to their widespread clinical applications. For example, although iPSCs now can be generated without a genome integrating approach, i.e. miRNA, episome, sendai-viral, mRNA and small molecules, the efficiency of iPSCs production remains relative low (~0.1%)[4, 5]. In addition, the efficiency of differentiation of iPSCs to the desired cell lineage varies among different iPSCs lines. It remains unclear which particular somatic cell sources are preferable for reprogramming.

Among the four reprogramming factors, KLF4 and c-MYC could be replaced by other factors. In contrast, OCT4 and SOX2 are thought to be essential for induction and maintenance of pluripotent identity. Although it has recently been discovered that mesendodermal and ectodermal lineage specifiers can induce pluripotency in the absence of both OCT4 and SOX2 [6, 7], the mechanisms by which lineage specifiers affect reprogramming remain elusive. There is no consensus on the selection of the cell sources for reprogramming to iPSCs [1, 8–14] or on which particular donor cell type has a higher efficiency of specific cell-type differentiation from its corresponding iPSCs [15]. Thus, the varying efficiencies in reprogramming of different somatic cell sources suggests that cell origin be an influence. Recent studies have further demonstrated that iPSCs may differentiate towards their cell-of-origin. The identification of appropriate adult somatic cell types with favorable properties for pluripotency reprogramming and preferred lineage differentiation, and definition of their advantages and disadvantages are therefore of high clinical value. Here, we reported that human adult conjunctiva epithelial cells (OECs) with endogenous expression of OCT4 and SOX2 can yield high efficiency on iPSCs generation (OECiPSCs) when using a protocol with four-reprogramming factors. Compared with ocular stromal cell-generated iPSCs (OSCiPSCs), OECiPSCs display higher efficiency for retinal pigmented epithelial cell differentiation.

## Materials and Methods

### Isolation of conjunctival epithelial cells (OECs) and conjunctival stromal cells (OSCs)

Collection of conjunctival tissues was approval by Clinical Research Ethical Review Board of the Medical College of Xiamen University (Permit Number: 20090412–1). For protection of personal data, ethical review board approved acceptance of patients’ verbal consents to use discarded conjunctival biopsies following eye surgery, documented in the patients’ medical records. The discarded samples from three donors were documented with code numbers only and personal data (i.e., full name/ID) were collected. Epithelial tissues and stromal tissues were separated aseptically under a stereo microscope (Olympus S2X2-ILLT) in the AireGard Horizontal Laminar Airflow Workstation (NuAire, Cat. No. NU-201-230E). Tissues (both epithelial and stromal) were cut to pieces approximately 3x3 mm^²^. Epithelial tissue was cultured in Keratinocyte Serum-Free Medium (KSFM). (Cat. No. 17005–042, Life Technologies). Stromal tissue was cultured in Stromal Cell Culture Medium (SCCM). A monolayer of conjunctival epithelial cells (OEC) or conjunctival stromal cells (OSC) emerged from the corresponding tissues within a week. We kept our passage number <5 in both cultures. Formulation of SCCM was as follow: Dulbecco’s Modified Eagles Medium (DMEM) (Cat. No. SH30022.01, Thermo Scientific); 10% Fetal Bovine Serum (FBS) (Cat. No. 16000–044, Life Technologies); 100 U/ml Penicillin-Streptomycin (P/S) (Cat. No. 15140–122, Life Technologies); 100 U/ml Amphotericin B (Cat. No. 1397-89-3, Sigma). Information about the ocular samples and their corresponding iPSCs is provided in S1 Fig.

### Immunofluorescence staining and Western Blotting

Standard immunochemistry staining and Western blotting were performed according to previous descriptions [16]. Briefly, ocular sections were embedded in paraffin wax, de-waxed, antigen unmasked and stained with a DAB kit (Cat. No. ab64238, Abcam) according to the manufacturer’s instructions. For standard immunofluorescence staining, cells were fixed in 4% paraformaldehyde for 30 minutes, washed, blocked and permeabilized in blocking solution. Cells were incubated with primary antibodies in blocking solution at 4°C overnight, washed twice and incubated with the corresponding secondary antibodies (Cell Signaling Technology) for 1 hour at room temperature. Cells were washed twice and stained with DAPI (Sigma) for 5 minutes, and then observed and photographed using a LEICA DMI6000B microscope (Leica Microsystems GmbH). Standard Western blotting was performed as previously described [16]. Membranes were developed using Immobilon Western Chemiluminescent HRP Substrate (Cat. No. P36599A, Millipore). (Antibodies used in immunochemistry and immunofluorescence and Western blotting were listed in S2 Fig).

### RNA isolation, reverse transcription, semi-quantitative PCR and real-time PCR

Total RNAs from iPSCs lines were extracted using a RNA Mini Kit (Cat. No. 74104, Qiagen). Reverse transcription was performed using 0.5 μg RNA in a final volume of 20 μl, using Primescript RT reagent kit (Cat. No. HRR047 A, TaKaRa), according to the manufacturer's instructions. Details of a panel of pluripotent genes and their corresponding primers are summarized in S3 Fig. Semi-quantitative reverse transcriptase PCR was performed with the designed primers of the targeted genes (S3 Fig) by Pfu DNA polymerase kit (Cat. No. M7741, Promega) as per the manufacturer's protocol. The PCR conditions were 95°C for 1min; 30 cycles of 95°C for 30 secs, annealing temperature 42°C for 30 secs, and 72°C for 2 mins; and a final extension at 72°C for 5mins. Beta-actin was used for normalization and all items were measured in triplicates. RT Real-time PCR was performed with the designed primers of the targeted genes (S4 Fig) by RT Master Mix (Cat No. HRR036A, TaKaRa) and CYBR Green Mix (Cat No. HRR820A, TaKaRa) as listed in manufacturer’s protocols. Real-time PCR was performed with Real-time PCR system (Step-one Plus, Applied Biosystems, Life Technologies).

### Generation of iPSCs from conjunctival stromal cells (OSCs) and conjunctival epithelial cells (OECs)

OSCs and OECs were maintained in SCCM and KSFM respectively. Two successive rounds of infection were performed as described [15]. Briefly, cells were infected with viral supernatant generated by transfection of 293T cells with retroviral PMXs vectors (Addgene) containing the cDNAs of human *KLF4*, *cMYC OCT4* and *SOX2*. After the second round of infection, transduced cells were cultured in iPSCs medium. Infection efficiency was separately monitored. As demonstrated by GFP- transduced expressing cell, the efficiency of our iPSCs generation was close to 100% (S5 Fig). On day 3, 50,000 cells were seeded onto a layer of feeder and maintained with iPSCs medium (Life Technology, Cat No: A1412901). On day 7, the culture was maintained in iPSCs medium in addition with the addition of valproic acid (VPA; 1mM) (Cat No. 1069665, Sigma). On Day 10, the medium was changed to iPSCs medium without VPA. On Day 10–20, colonies that were sufficiently large and resembling human ESCs (i.e. flat morphology with defined borders and big nuclei containing prominent nucleoli) were selected mechanically and expanded in human iPSCs medium on feeders as described previously [15].

### Alkaline phosphatase staining

Briefly, culture was fixed with 4% paraformaldehyde for 20 mins, washed with PBS and stained with alkaline phosphatase for 0.5–2 hrs. Excess stain was removed with PBS and the culture viewed under light microscope.

### Characterization of human iPSCs

To characterize the iPSC lines, standard procedures were performed: 1) immunostaining for pluripotent markers; 2) teratoma formation; 3) H&E staining; 4) karyotyping and 5) bisulfate conversion-pyrosequencing.

For immunofluorescence staining, the standard procedures were used as described in Methods and Materials. Primary antibodies used in the staining were listed in S2 Fig. The corresponding secondary antibodies were purchased from Cell Signaling Technologies.

For hematoxylin/eosin (H&E) staining on teratomas, iPSCs were injected subcutaneously or intramuscularly into the right hind leg of immunocompromised NOD/SCID mice. Teratomas were excised after 8 weeks, fixed, embedded in paraffin, sectioned and stained with hematoxylin / eosin.

For karyotyping, standard G-banding chromosome analysis was carried out as previously described [16]. Briefly, the iPSCs were trypsinized and fixed in 3:1 methanol/acetic acid. The fixing procedure was repeated three times. Finally, the pellet was suspended in a final volume of 1.5 ml of fixative, and the cells dropped onto glass slides. Metaphases of cells were G-banded and karyotyped in accordance with the International System for Human Cytogenetic Nomenclature recommendations (1995). 30 metaphases were analyzed and 3 were fully karyotyped for this cell line using a conventional microscope and the Goodline-software (Beijing).

For Bisulfite conversion and pyrosequencing, OECs, OSCs and IMR90 and their corresponding iPSCs were harvested for bisulfite and pyrosequencing as follows. Bisulfite conversion and pyrosequencing were done according to the methods described in 2007 Nature Protocols[22]. Briefly, in three parts: (Part 1) bisulfite treatment, (Part2) amplifying the regions of interest in the bisulfite conversed samples by PCR and (Part3) pyrosequencing. (Part1) Bisulfite treatment. 1mg DNA was bisulfite converted treated with CpGenome Fast DNA Modification Kit (Millipore Cat. No. S7824) according to manufacturer’s instructions. (Part 2) The region of interest in the bisulfite-converted samples was amplified by PCR using AmpliTaq Gold 360 Master Mix (Life Technologies, Cat. No. 4398881) according to listed in manufacturer’s instructions. Positive and negative controls were included. The PCR was placed in a thermal cycler (Applied Biosystems, Gene Amp PCR systems 9700, 96-Well Gold-Plated; Cat. No. 4314878) activating the polymerase by incubating at 95°C for 15 min. The amplification reaction was carried out for 30 cycles with 30 sec denaturation at 95°C, annealing at approximately 60°C for 30 sec, and extension for 10 sec at 72°C. Only single, strong bands of amplified products were further processed. Biotin-labeled amplification primers were listed in S6 Fig (Part 3) Pyrosequencing. Samples were sent to Genome Center of The University of Hong Kong, for Pyrosequencing Service using the Qiagen pyrosequencing system and softwares (Biotage-Qiagen PSQ 96MA). The sequence runs were analyzed by the Q-CpG software. CpG islands were listed in S7 Fig.

### Microarray and data analysis

For each sample, OEC1, OEC2, OSC, OEC1iPSC, OEC2iPSC, OSCiPSC and hESC, we performed a microarray with Affymetrix Human Genome U133 plus 2.0 arrays on two replications. Total RNA was extracted from cultured cells using the RNeasy Mini Kit (Cat. No. 74104; Qiagen, Valencia, CA). Five micrograms of total RNA was used for microarray hybridization. The fragmented complementary RNA was hybridized with the Human Genome U133 plus chips (Affymetrix, Santa Clara, CA), using the Hybridization Oven 640 (Affymetrix). The washing and labeling procedures were performed using the Fluidics Station 400 (Affymetrix) according to the manufacturer's instructions. The arrays were scanned using the GeneChip Scanner 3000 (Affymetrix), and the signal intensity for each transcript was determined using Microarray Suite Software 5.0 (Affymetrix). Statistical data analysis was carried out using software JMP (SAS, North Carolina).

The top 20 genes preferentially up-regulated in OECs were investigated by the DAVID Bioinformatics Resources 6.7. Enrichment analysis was based on the DAVID recommended functional annotations, including OMIM disease, sequence feature, Gene Ontology, KEGG pathway, protein domain, etc. When members of two independent groups could fall into one of two mutually exclusive categories, the Fisher Exact test was applied to determine whether the proportions of those falling into each category differed by group. In the DAVID annotation system, the Fisher Exact was adopted to measure the gene-enrichment in annotation terms. The background here is all the 23483 genes Affymetrix Human Genome U133 plus 2.0 arrays. The EASE Score and a modified Fisher Exact P-Value were obtained for each annotation term involved: the smaller the EASE Score, the more the particular gene was enriched. Each enrichment score of the particular individual gene group stands for the geometric mean of negative denary logarithm of EASE scores of those terms involved in the particular group.

### Differentiation of human iPSCs

To determine the in vitro differentiation potential of OECiPSCs, OSCiPSCs and iMR90iPSCs, the iPSCs were subjected to sphere culture for 7 days followed by monolayer culture for 3 days. Cells were harvested for testing endodermal, mesodermal and ectodermal markers. For ocular surface epithelial differentiation, the iPSCs colonies were grown on collagen IV coated plates cultured with SHEM medium for 2 days followed by KSFM medium for 7–21 days until epithelial-like cells emerged from the differentiating OECiPSCs. SHEM formulation: DMEM/F12 (Cat. No. Hyclone SH30023.01) FBS (Cat. No.16000-044, Life Technologies) 5% HEPES (Cat. No.151630-080, GIBCO) Insulin-Transferrin-Selenium (ITS; 100x) (1%) (Cat. No. 51500–056, GIBCO); (100x) Hydrocortisone (0.5 μg/ml) (Cat. No. 0025012ID, Life Technologies); Dimethyl sulfoxide (DMSO; 0.5%) (Cat. No. 67685, Sigma); EGF (2ng/ml) (Cat. No. PHG0311, Life Technologies). For retinal pigmented epithelial differentiation, we used an ‘‘retinal determination” protocol typically resulting in cultures with 80% retinal cells in the presence of insulin growth factor 1 (IGF1), Dickkopf-1(DKK-1) and Noggin [15–17]. Immunofluorescence staining of the differentiated cells from IMR90, OSCs and OECs was performed with the standard protocol as described previously in our Methods and Materials. The markers used in this step are listed in S2 Fig. The corresponding secondary antibodies were purchased from Cell Signaling Technology.

### Statistical analysis

Data were expressed as mean ± standard deviation (SD). The significant differences between groups were analyzed with unpaired Student *t-*test for the two groups, or one-way ANOVA followed by Bonferroni test for more than 2 groups. All data was analyzed with SPSS10.0 software package for Windows (SPSS Inc., Chicago, IL). A value of *P*<0.05 was considered statistically significant.

## Results

### Expression of pluripotency reprogramming factors in conjunctival epithelial cells from human conjunctival tissue explants

**Fig 1:**
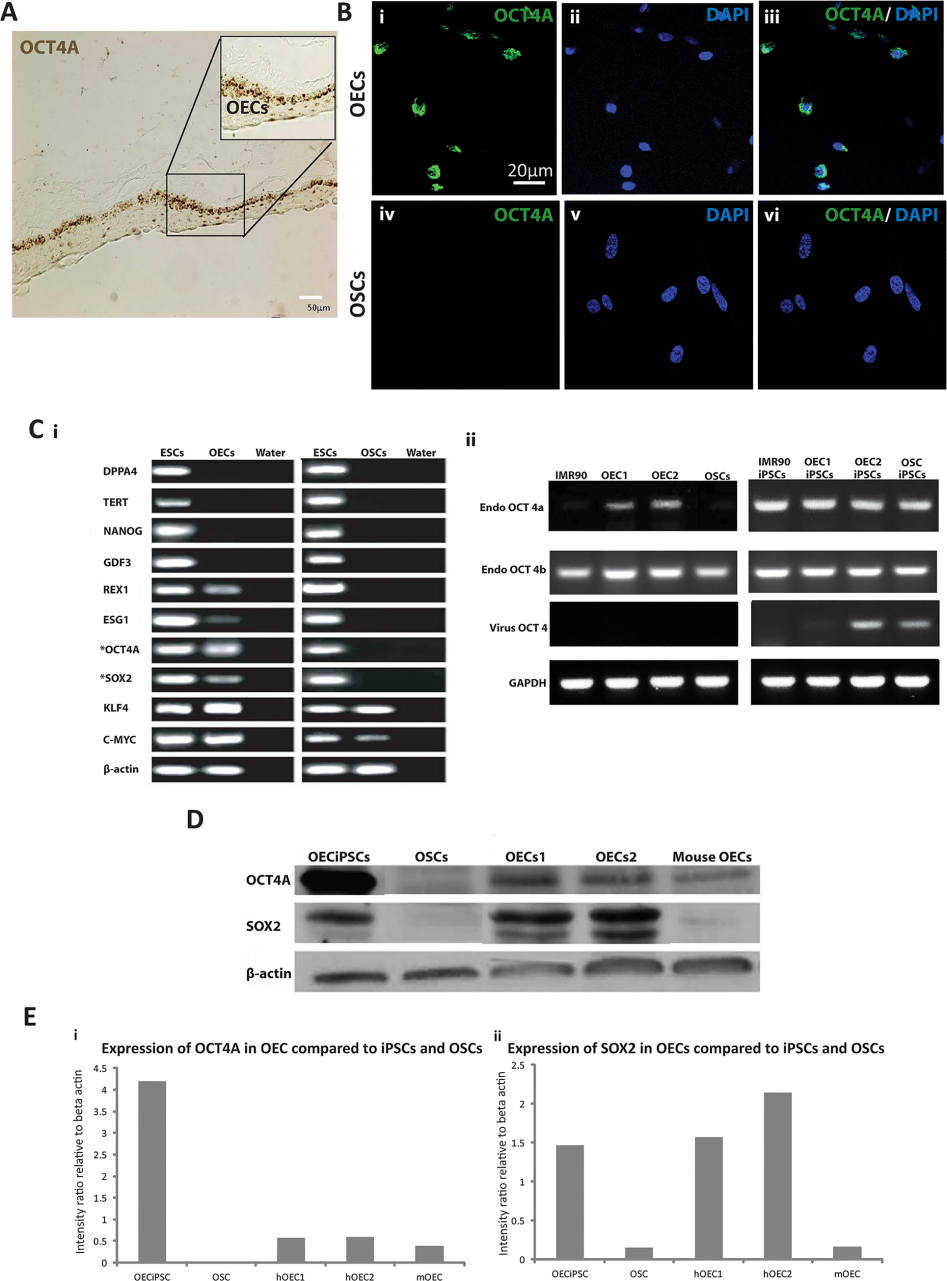
Endogenous expression of OCT4A and SOX2 pluripotency reprogramming factors in OECs derived from conjunctival tissues. **(A)** DAB-based immunohistochemistry staining displayed the expression of OCT4A (brown, arrows) in human ocular sections. OCT4A expressed in the epithelium layer but not in stromal layers (OEL, ocular epithelial layer; OSL, ocular stromal layer). **(B)** Immunofluorescence staining demonstrated OCT4A expression in OECs but not in OSCs. **(C) (i)** Reverse transcription polymerase chain reaction (RT-PCR) results in expression of pluripotency genes (from top to bottom Panels): *DPPA4*, *TERT*, *NANOG*, *GDF3*, *REX1*, *ESG1*, *OCT4A*, *SOX2*, *KLF4* and *C-MYC* and *Beta- actin*. Human ESCs and water were included as positive and negative controls respectively. **(ii)** Expression of OCT4 isoforms OCT4A and OCT4B, endogenous OCT 4 and viral OCT4 detecting using 5’ and 3’ UTR sequences tracking with RT-PCR. (From left to right) Primary lines (IMR90, OEC1, OEC2 and OSCs) and their corresponding iPSCs. **(D)** Western Blotting for OCT4A and SOX2 protein levels in OECs, OSCs and iPSCs. Lane 1: OECiPSCs, Lane 2: OSCs, Lane 3&4: OEC1&2 respectively, Lane 5: mouse OECs **(E)** Expression levels of (i) OCT4A and (ii) SOX2 were represented in intensity ratios respectively.

We recently isolated conjunctival epithelial cells (OECs) and conjunctival stromal cells (OSCs) from individual conjunctival specimen in conditioned cultural medium (S5 Fig). Using a characterized antibody (Santa Cruz Biotechnology, CA. Cat No: SC-8626) that specifically targeted the pluripotency factor OCT4A [17], we detected OCT4A expression mainly in the basal layer of conjunctival epithelium, but not in the stromal cell layers (Fig 1A). Immunofluorescence staining also disclosed that OCT4A is expressed mostly in conjunctival derived OECs, not in OSCs (Fig 1B). Similarly, pluripotency reprogramming factors, OCT4A and SOX2 are highly expressed in the basal layer of conjunctival epithelium rather than the stromal cell layers (Fig 1Ci). This is consistent with previous reports that OCT4 and SOX2 are expressed in corneal and conjunctival epithelium [18–20] In addition, isoform OCT4B was found widely expressed in OECs, OECiPSCs, OSCs, OSCiPSCs, skin fibroblast IMR90 (ATCC, CCL-186) and IMR90iPSCs (Fig 1Cii). Retrovirus-mediated reprograming factor OCT4 can be detected in OECiPSCs and OSCiPSCs, but not in OECs, OSCs and IMR90 somatic cells. (Fig 1Cii; S3 Fig for primers). Western blotting revealed expression of OCT4A in both human and mouse OECs at approximately 10%-15% of the protein level found in iPSCs (Fig 1D and 1Ei). Meanwhile, another reprogramming factor, SOX2, was also detected in mouse and human OECs (Fig 1D and 1Eii).

Compared with OSCs, RT-PCR confirmed a higher expression of some pluripotency genes (e.g., *REX1*, *ESG1*, *OCT4A*, *SOX2*, *KLF4 and c-MYC*) in OECs (Fig 1Ci). Notably, only *OCT4* and *SOX2* were expressed in embryonic stem cells (ESCs) and OECs, and were absent in OSCs (Fig 1Ci, * marked). Furthermore, *KLF4* and *c-MYC* were also present in OECs in addition to *OCT4* and *SOX2*. Nonetheless only *KLF4* and *c-MYC* were identified in OSCs (Fig 1Ci). The endogenous expression of reprogramming factors in OECs provides important clues that OECs may offer a favorable somatic cell source for the generation of iPSCs.

### Efficiencies of human iPSCs derived from conjunctival epithelial cells (OECs) and conjunctival fibroblasts (OSCs)

We examined the susceptibility of these two cell types to retroviral transduction; GFP-encoding retroviral supernatant was used to infect OECs and OSCs obtained from an individual in a single biopsy. No significant differences were observed in the percentage of transduced cells (indicated by the percentages of GFP-positive cells) or their corresponding median intensity of GFP fluorescence (S5 Fig). Thus retroviral transduction efficiency in this study did not explain differences in reprogramming efficiencies among OECs and OSCs.

**Fig 2:**
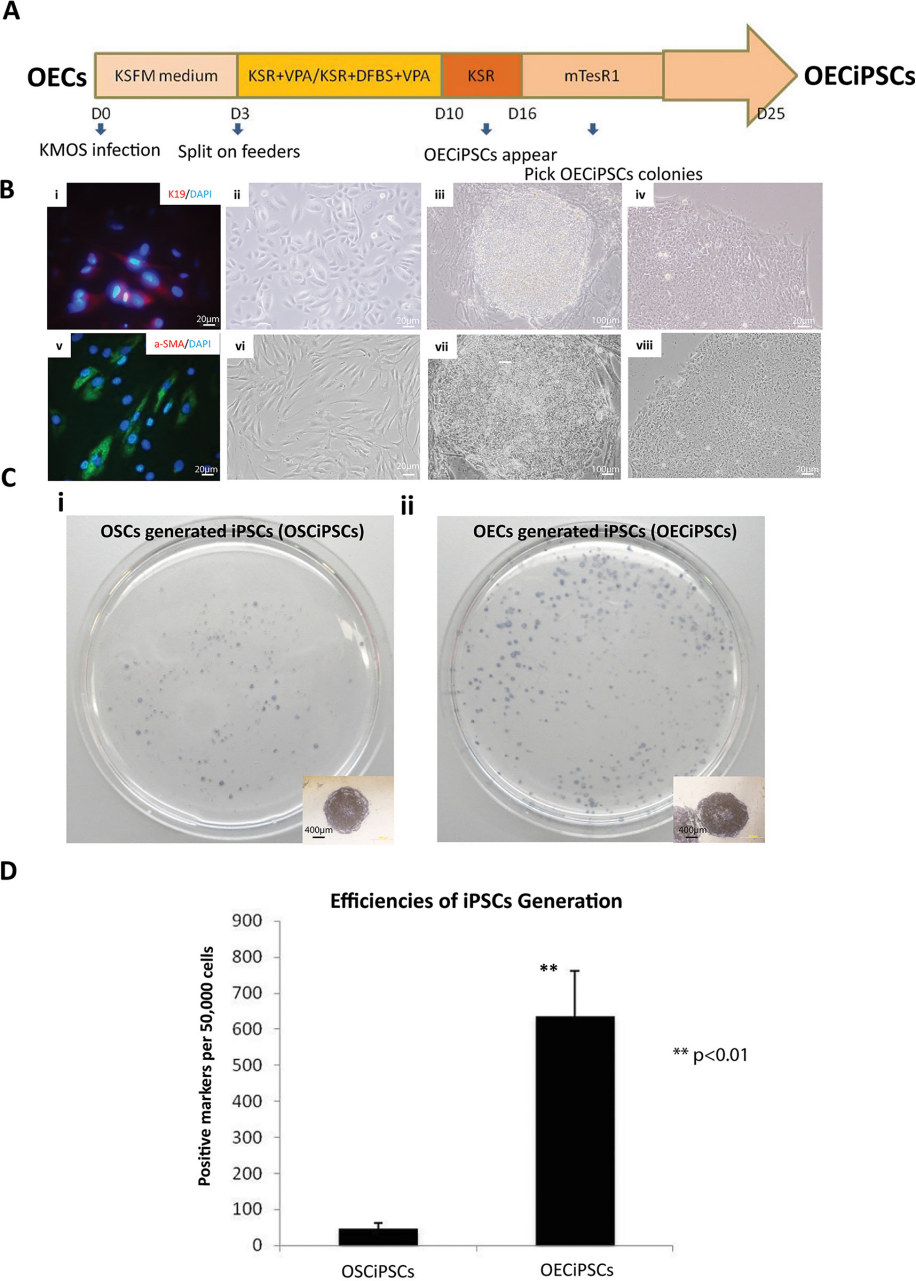
Efficiency of human iPSCs generation from OECs. **(A)** Schematic representation on human iPSCs generation from human OECs. **(B)** Bright view images on iPSCs generation **(i)** OECs primary cells **(ii)** OECiPSCs on feeders **(iii)** OECiPSCs on feeder free culture. **(C)** Alkaline phosphatase (AP)-staining for iPSCs-like colonies derived from **(i)** OSCs & **(ii)** OECs. **(D)** Quantitative analysis of AP-positive iPSC-like colonies between OSCiPSCs and OECiPSCs OSC-iPSCs vs OEC-iPSCs: (46.33±15.50; 635±127.57) (SD; N = 3, **P<0.01).

To test the timing of reprogramming OECs and OSCs by the four factors, the same retroviral supernatant was used for the transduction of primary OECs and OSCs. One day following infection, OECs and OSCs culture was changed to culture in ESCs medium and maintained on mouse stromal feeders for iPSCs generation as previously described[15, 16]. Morphologically, nascent hESCs-like colonies could be identified in OECs culture as early as 10–12 days post-infection, (Fig 2A & 2Bii). This was approximately 5–7 days earlier than our OSCs culture and 10 days earlier than skin stromal cells[21].

To test the efficiency of reprogramming OECs and OSCs by the four factors, Alkaline Phosphatase(AP) staining was performed to count the generated iPSCs colonies from 50,000 loaded for OECs or OSCs cultures respectively. To do this, the same retroviral supernatant was used to transduce primary OECs and OSCs. We obtained ~760 OECiPSCs colonies (652 ± 104, N = 3) from ~50,000 infected OECs (Fig 2C and 2D). The reprogramming efficiency to iPSCs was quantified based on cell morphological criteria and Alkaline Phosphatase-positive (AP^+^) staining. OECs displayed an overall reprogramming efficiency close to 1.5%. For OSCs, infection was done in parallel. Around 100 granulated colonies and ~50 hESC–like AP^+^ colonies (46 ±15, N = 3; Fig 2C and 2D) were obtained at the 30^th^day post-infection from ~50,000 infected OSCs, giving an overall efficiency up to 0.1% (Fig 2D, **P<0.01).The efficiency of iPSCs generation from OECs (1.5%) is higher than that of our OSCs (0.1%) and the karotinocytes (1%) from the previous report [10], on the same ~50,000 starting primary cells for reprogramming. In addition, efficiency of OSC reprogramming was at least 10-fold higher than that of skin stromal cell reprogramming that ranged from 0.001%-0.01% [22]. These results show that OECs are more amendable to pluripotency reprogramming than most other cell types, e.g. OSCs.or skin stromal cells.

### Characterization of pluripotency and comparison of gene expression profiles of the generated OECiPSCs and OSCiPSCs

**Fig 3:**
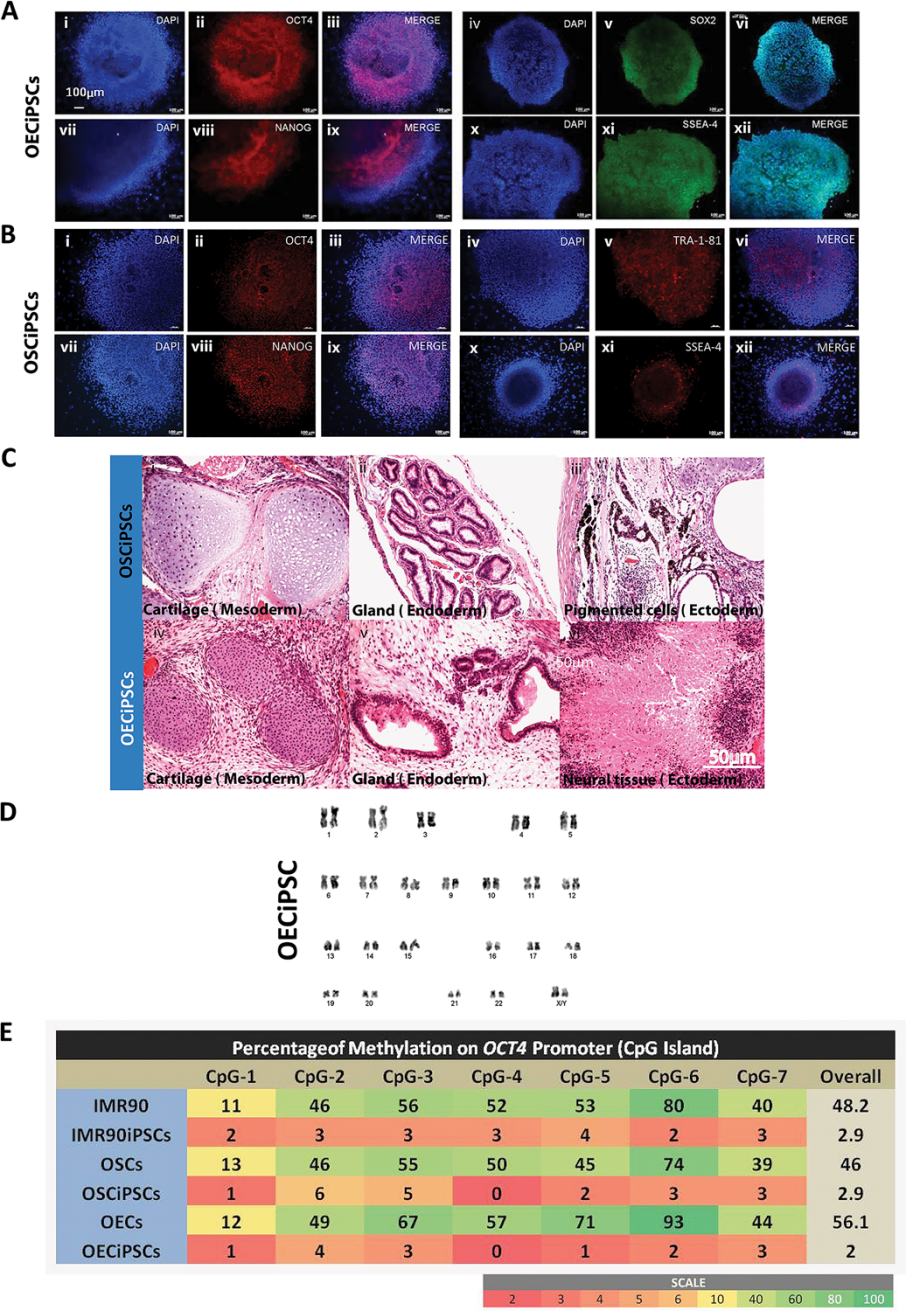
Characterization of pluripotency of OECiPSCs and OSCiPSCs. **(A)** Immunostaining of pluripotency markers in OECiPSCs. **(i-iii)** OCT4, **(iv-vi)** SOX2, **(vii-ix)** NAGONG and **(x-xii)** SSEA-4. **(B).** Immunostaining of pluripotency markers in OSCiPSCs. **(i-iii)** OCT4, **(iv-vi)** TRA-1-81, **(vii-ix)** NANOG and **(x-xii)** SSEA-4 **(C)** Hematoxylin and eosin staining of teratoma tissues harvested from SCID mice after OECiPSCs and OSCiPSCs injection. Teratomas from both OECiPSCs and OSCiPSCs were constituted with mesodermal, endodermal and ectodermal germ-layer cell types, including cartilage, gland and pigmented cells/ or neural cells. **(D)** Karyotypes of representative iPSCs, OECiPSCs, was analyzed and normal chromosome structures were observed (OSCiPSCs chromosome structures similar to OECiPSC, data not shown).**(E)** Bisulfite conversion pyrosequencing data showing the percentages of methylation on *OCT4* promoter of the primary cells (IMR90, OSCs and OECs) verus their corresponding iPSCs (IMR90iPSCs, OSCiPSCs and OECiPSCs).

Several iPSCs-like colonies generated from OECs and OSCs were selected and expanded, and cell lines established for pluripotency characterization. Pluripotency markers OCT4, NANOG, SOX2, SSEA-4 and TRA-1-81 were positively detected by immunofluorescence staining in both OECiPSCs and OSCiPSCs (Fig 3A and 3B respectively). When these iPSCs lines were subcutaneously injected into immune deficient SCID mice, teratoma formation was observed around 8–12 weeks post-injection of both OECiPSCs and OSCiPSCs. Sectioning of representative teratomas revealed that the tissues constituted mesodermal, endodermal and ectodermal germ-layer cell types, including cartilage, gland and pigmented cells/ or neural cells (Fig 3C) in both OECiPSCs and OSCiPSCs. Karyotyping analysis of OECiPSCs line revealed normal chromosomal structures (Fig 3D). Another hallmark of successful reprogramming of somatic cells into pluripotent stem cells is the low methylation of pluripotent genes that are hypermethylated in differentiated cells. To this end, we analyzed the well-characterized *OCT4* promoter region that was highly methylated in OECs, OSCs or IMR90 but became demethylated in their corresponding iPSCs lines. Our data in methylation study demonstrated successful epigenetic reprogramming of somatic cells to pluripotent status (Fig 3E and S3 and S7 Figs). One interesting finding is that OECs endogenously express higher OCT4 but also higher methylation on its promoter in OECs as compared to OSCs or IMR90 fibroblast; it suggests expression level of gene is not always parallel to methylation status. This phenomena had been reported that gene expression is not always inversely correlated with the methylation regulation on CpGs promoter [23].

**Fig 4:**
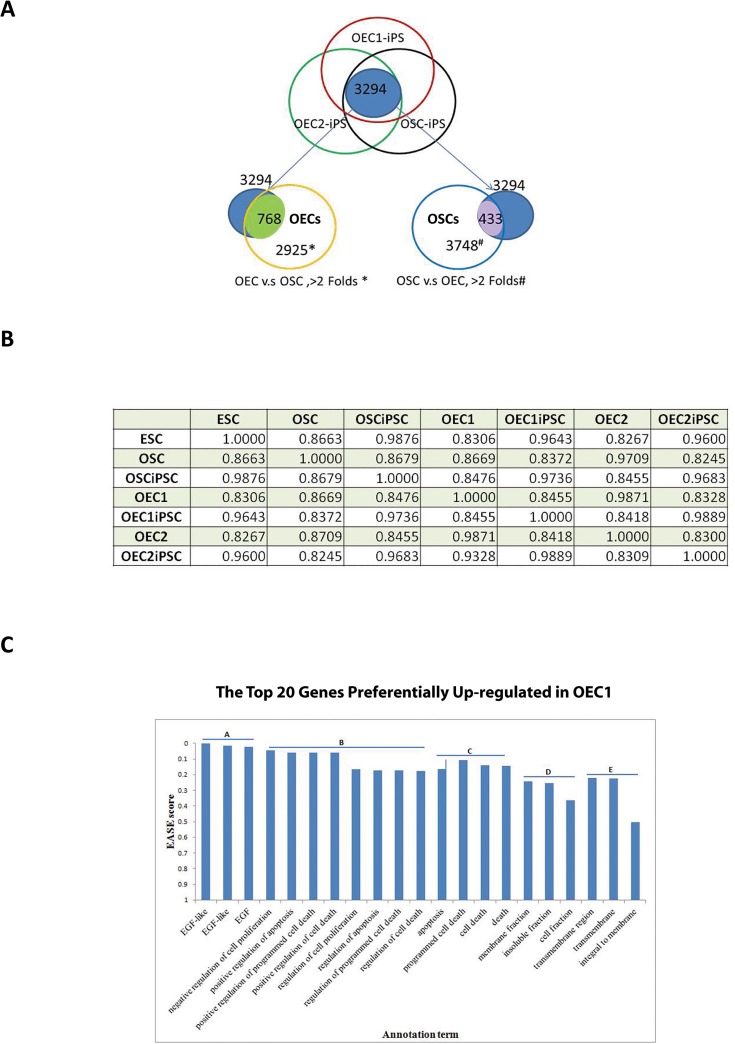
Pairwise comparison of global gene expression among different cells. **(A)** 3249 different genes were preferentially expressed among the iPSCs lines (IMR90iPSCs, OSCiPSCs and OECiPSCs). Among those 3249 genes, there were 768 genes preferentially presented in OECs when compared to OSCs above 2-fold cutting-off difference. There were only 433 genes preferentially presented in OSCs when compared with OECs above 2-fold cutting-off difference. **(B)** Correlation coefficients between different cell types in genome-wide transcriptional profiles were listed among hESC, OSC, OSCiPSCs, OEC1, OEC1iPSCs, OEC2, OEC2iPSCs. **(C)** The top 20 genes preferentially up-regulated in OEC1 vs. OSC were classified into biological processes using the NIH DAVID Pathway Analysis. Data for OEC2 vs OSC is presented in S8 Fig.

We next compared genome-wide transcriptional profiles of OECs, OSCs, and hESCs with their respective iPSCs. There are 2925 of 23483 genes found >2 folds in OECs compared with OSCs, and 7811 out of 23483 genes were observed above >0.5 and <2 folds in OECs. On the other hand, OEC1iPSCs, OEC2iPSCs and OSCiPSCs shared 3249 genes (with coefficient of variation less than 300). Among the 3249 genes, 768 genes were preferentially expressed in OECs (>2 folds, comparing OECs with OSCs), but only 433 genes were preferentially expressed in OSCs (>2 folds, comparing OSCs with OECs) (Fig 4A). These suggest that, OECs express more of these common genes of iPSCs than OSCs. This data may provide some molecular explanation of why OECs obtain pluripotency easier than OSCs on ‘KMOS’ reprogramming.

Pairwise comparisons of OSCs vs OSCiPSCs, and OEC (1&2) vs OEC (1&2)-iPSCs confirmed the distinction of somatic cells and iPSCs with low correlation coefficients of 0.868 and 0.846 & 0.831 respectively (Fig 4B). In contrast, the correlation coefficients of OSCiPSCs, OEC1iPSCs, and OEC2iPSCs with the same reference ESCs (H9) were virtually identical at 0.988, 0.964, and 0.960, respectively (Fig 4B). This suggests that OSCiPSCs and OECiPSCs are very similar to ESCs. Pairwise comparison of global gene expression by OEC1 and OSCs revealed a correlation coefficient of 0.867 (Fig 4B). This result suggests that despite shared expression of common genes, there are also significant differences between OECs and OSCs.

The top 20 genes preferentially up-regulated in OECs were classified into biological processes using the NIH DAVID Pathway Analysis [22, 24]). It revealed that OECs are tightly involved in the regulation of EGF signaling to maintain epithelial function, cell proliferation and apoptosis. All of these functions are crucial to cell reprogramming (Fig 4C and S8 Fig).

### Retinal pigmented epithelial differentiation and gene expression profiles of iPSCs lines generated from various cell origins

**Fig 5:**
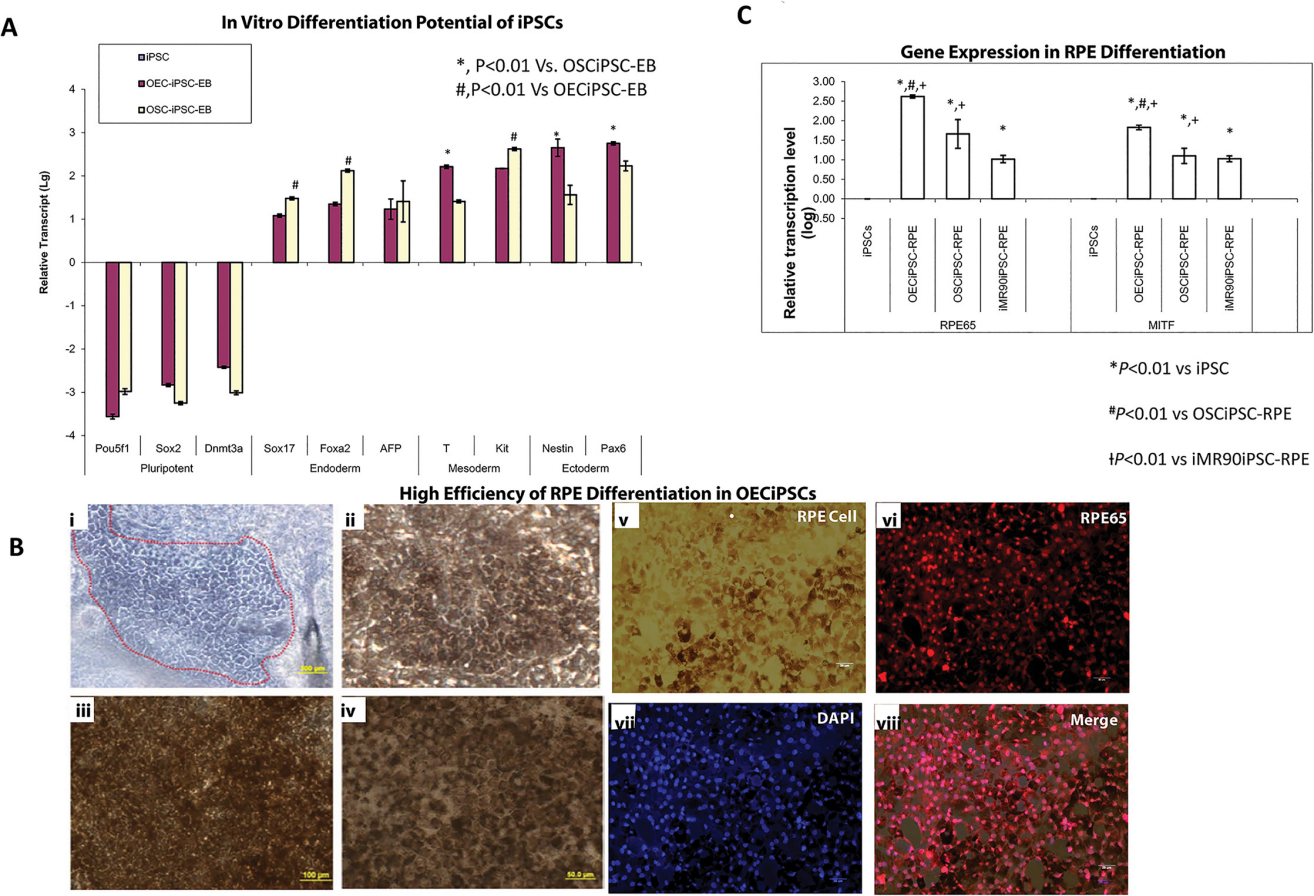
Ocular epithelial differentiation from different cell-of-origin iPSCs lines. **(A)** To compare the pluripotency nature of different types of iPSCs, OECiPSCs and OSCiPSCs were subjected to sphere culture for 7 days then monolayer culture for further 3 days. Cells were harvested for testing endodermal, mesodermal and ectodermal markers by real-time PCR. Expression level of pluripotency markers Pou5f1, Sox2 and Dnmt3a were dramatically decreased up to 1000 times in both differences in OECiPSCs and OSCiPSCs. The OSCiPSCs demonstrated a preference for endodermal differentiation while OECiPSCs show higher potential in ectodermal differ nation. iPSCs vs OECiPSCs vs OSCiPSCs: Pou5f1 (0.00±0;-3.56±0.06;-2.98±0.07); Sox2 (0.00±0;-2.83±0.04;-3.25±0.03); Dnmt3a (0.00±0;-2.42±0.03;-3.01±0.04); Sox17 (0.00±0; 1.08±0.04; 1.48±0.03); Foxa2 (0.00±0; 1.35±0.04; 2.12±0.03); AFP (0.00±0; 1.23±0.23; 1.41±0.47); T (0.00±0; 2.21±0.04; 1.41±0.03); Kit (0.00±0; 2.17±0.0; 2.62±0.03); Nestin (0.00±0; 2.65±0.20; 1.56±0.22); Pax6 (0.00±0; 2.75±0.04; 2.23±0.11); (SEM N = 3;*P<0.01vs OSCiPSCs-EB; ^#^ *P*<0.01vs OECiPSCs-EB). **(B)** (A) After 3 weeks of retinal differentiation protocol, the confined pigmented cells (black cells) were merged from differentiating OECiPSC clusters. (B-D) After manually picked up pigmented cell colonies into high-density cultures, the cells were able to re-acquire the morphology and pigmentation of RPE-like cells. (E-H) These pigmented cells were putative RPE-like cells as confirmed by immunoactivity for mature RPE marker, RPE 65 (DAPI/blue). **(C)** After 3 weeks, the increased expression level of RPE cell markers, RPE65 and MITF, were significantly different among OECiPSCs, OSCiPSCs and iMR90iPSCs under same RPE differentiation conditions. iPSCs vs OECiPSCs vs OSCiPSCs vs iMR90iPSCs: RPE65(0.00±0;2.62±0.04;1.66±0.37;1.02±0.09); MITF (0.00±0; 1.83±0.06; 1.1±0.19; 1.03±0.07). (Log SD; N = 3, *P<0.01 vs iPSCs; ^#^ *P*<0.01 vs OSCiPSCs-RPE;^Ɨ^P<0.01 vs iMR90iPSCs-RPE)

To compare the pluripotency of different types of iPSCs, OECiPSCs and OSCiPSCs were subjected to sphere culture for 7 days, then monolayer culture for further 3 days. Cells were harvested for testing endodermal, mesodermal and ectodermal markers by real-time PCR. The expression level of pluripotency markers Pou5f1 (OCT4), Sox2 and Dnmt3a were dramatically decreased up to 1000 times in both differentiating OECiPSCs and OSCiPSCs. The OSCiPSCs appeared to have a preference for endodermal differentiation and OECiPSCs showed higher potential in ectodermal differentiation (Fig 5A). After 3 weeks of retinal differentiation protocol, the confined pigmented cells (black cells) were merged from differentiating OECiPSC clusters. After manually separating pigmented cell colonies into high-density cultures, the cells were able to re-acquire the morphology and pigmentation of RPE-like cells. These pigmented cells were putative RPE-like cells, confirmed by their immunoactivity for mature RPE marker, RPE 65 (DAPI/blue) (Fig 5B). The highest gene expression level of RPE cell markers, RPE65 and MITF, was observed in OECiPSCs, compared to the differentiating OSCiPSCs and IMR90iPSCs under same RPE differentiation conditions (Fig 5C).

**Fig 6:**
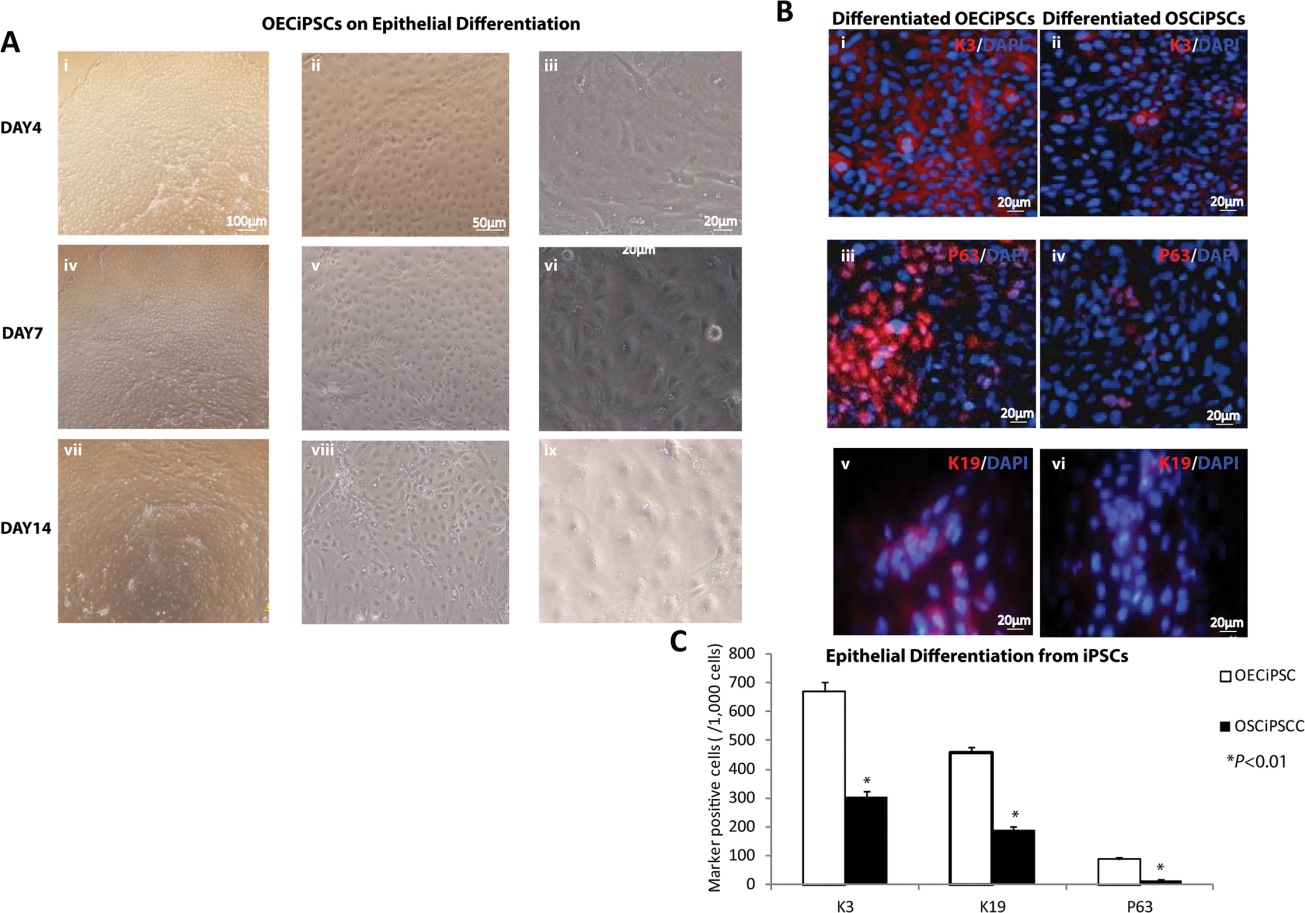
Efficiency of ocular epithelial differentiation in OSCiPSCs and OECiPSCs. **(A)** Epithelial differentiation of OECiPSCs under conditions of SHEM medium following by KSFM medium for 14 days. **(B)** Immunostaining revealed that K3, K19 and P63-positive cells were observed in OECiPSCs and OSCiPSCs. **(C)** Quantitatively analysis for K3, K19 and P63-positive cells showed that, compared to differentiated OSCiPSCs, differentiated OECiPSCs have more K3, K19 and P63-positive cells. OECiPSCs vs OSCiPSCs: K3 (68.6±5.9%.; 29.7±4.4%); K19 (48.0%±2.2%; 20.2%±1.0%); P63 (12.6%±1.1%; 2.5%±0.6%). (SD; N = 3;*P<0.01).

**Fig 7:**
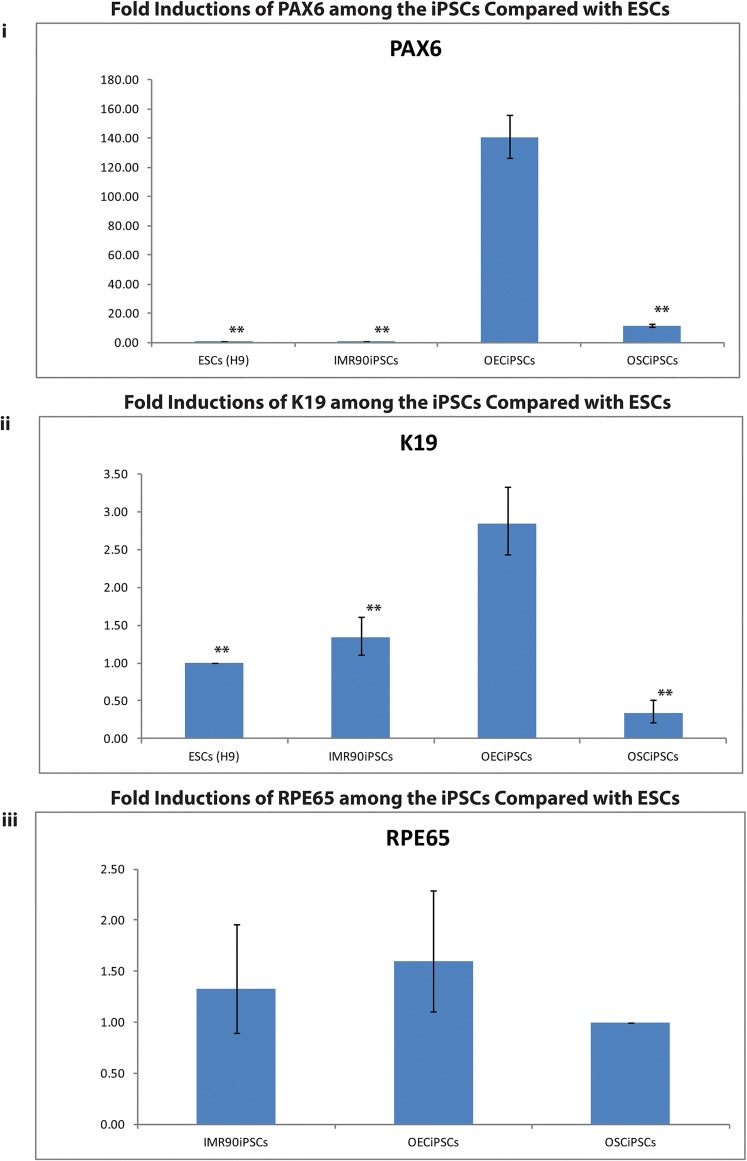
Ocular gene expression profiles among iPSCs. **(i&ii)** PAX6 and K19 profiles of OECiPSCs compared to ESCs, IMR90iPSCs and OSCiPSCs respectively. **(iii)** RPE65 profile of OECiPSCs compared to IMR90iPSCs and OSCiPSCs. (i) Data shows an upregulation of PAX6 (140.34 folds;*** p<0.01) compared with ESCs; and upregulations of PAX6 (11.97 and 131.56 folds, ***p<0.01) compared to OSCiPSCs and IMR90iPSCs respectively. ESCs (H9) vs iMR90iPSCs vs OECiPSCs vs OSCiPSCs: PAX6 (1.00±0.00; 1.067±0.12; 140.34±13.86;11.72±1.23) (SD; N = 3; ** *P*<0.005) (ii) Data shows an upregulation of K19 (2.85, 2.12 and 8.38 folds, ***p<0.01) compared to ESCs, IMR90iPSCs and OSCiPSCs respectively. ESCs (H9) vs iMR90iPSCs vs OECiPSCs vs OSCiPSCs: K19 (1.00±0.00; 1.34±0.23; 2.85±0.4137;0.34±0.1138) (SD; N = 3; ** P<0.005) (iii) The profile shows an upregulation of RPE65 (1.59 and 1.20 folds) compared to OSCiPSCs and IMR90iPSCs respectively. IMR90iPSCs vs OECiPSCs vs OSCiPSCs: RPE65 (1.33±0.43; 1.59±0.49; 1.00±0.00 (SD; N = 3; P>0.05).

Immunostaining for the epithelial differentiating iPSCs, under conditions of SHEM medium following with KSFM medium for 14 days (Fig 6A), revealed that K3, K19 and P63-positive cells in OECiPSCs and OSCiPSCs (Fig 6B). Quantitatively analysis of 1000 cells K3, K19 and P63-positive cells showed that, compared to differentiated OSCiPSCs, differentiated OECiPSCs have more K3, K19 and P63-positive at 68.6± 5.9% v.s 29.7± 4.4%, 48.0± 2.2% v.s 10.2± 1.0%, and; 12.6± 1.1% v.s 4.5± 0.6% respectively (Fig 6C). In addition, we performed immunostaining against K19, P63 and RPE65 in OECiPSC-induced teratoma sections. Abundant K19-positive cells, P63-positive cells and RPE65-positive cells (retinal pigmented epithelial marker) were detected, and generally dispersed throughout the tumor tissue sections (S9 Fig). Beneficial upregulation of *PAX6* expression was observed in OECiPSCs for ocular cell lineage differentiation. Gene expression profiles on selected ocular genes were tested among OECiPSCs, OSCiPSCs, ESCs and IMR90iPSCs (S10 Fig and Fig 7).

Analysis from microarray data revealed 8.04 and 77.61-fold increased expression of *PAX6* in OECiPSCs when compared to OSCiPSCs and ESCs respectively (S10 Fig). When compared with OSCiPSCs, OECiPSCs showed 4.82, 2.29 and 2.27 fold regulation of *RPE65*, *COL3A1* and *SOX2* expression respectively (S10 Fig). When compared with ESCs, OECiPSCs showed 8.58 and 4.18folds upregulation of *COL3A1* and *SOX2* expression (S10 Fig). To validate the above microarray data, we performed RT real-time PCR on selected ocular genes (Fig 7). We confirmed upregulation of *PAX6*, *RPE65* and *K19* genes in OECiPSCs compared with ESCs, OSCiPSCs and IMR90iPSCs. For instance, *PAX6* level of OECiPSCs was upregulated by 140.34 folds that of ESCs, *PAX6* level of OECiPSCs is upregulated by 11.97 and 131.56 fold than that of OSCiPSCs and IMR90iPSCs respectively (Fig 7i). For *RPE65* expression level, OECiPSCs showed an upregulation of 1.59 and 1.20 fold when comparing to OSCiPSCs and IMR90iPSCs respectively (Fig 7iii). Furthermore, OECiPSCs showed upregulation in *K19* expression with 2.85, 2.12 and 8.38 fold increase compared to ESCs, IMR90iPSCs and OSCiPSCs respectively (Fig 7ii). Combined microarray and RT real-time PCR analysis revealed a high basal level of *PAX6* and other ocular –related genes (*RPE65*, *COL3A1* and *K19*) in OECiPSCs compared with other iPSCs, suggesting OECiPSCs are a promising iPSCs source for ocular epithelial differentiation.

## Discussion

In our study, ocular epithelial cells (OECs), with expression of endogenous reprogramming factors OCT4 and SOX2, provide a very attractive somatic cell source for highly efficient iPSCs generation. In addition, OECiPSCs displayed stronger potentials for ocular epithelial differentiation (i.e. RPE cells) than OSCiPSCs, which may be associated with high basal level of PAX6, an essential element for ocular development. Cell origin is found to influence iPSCs generation although the underlying mechanisms are not fully understood. It has been suggested that somatic cells in an advanced state of reprogramming tend to have superior reprogramming efficiencies, and the corresponding reprogramming status is reflected by the endogenous expression of pluripotent factors [25]. For example, compared with terminal differentiated somatic cells (e.g., T lymphocytes) and progenitor cells (e.g., neonatal cord blood stem cells and neural progenitor cells) are more easily reprogrammed to pluripotency. Only the introduction of *OCT4* or/and *SOX2* is sufficient to induce pluripotency in these cell sources [11]. In contrast, four factors are required for reprogramming terminal T cells to pluripotency [26]. Nonetheless, the availabilities of neonatal cord blood stem cells and neural stem cells are limited in adults. Among adult somatic cells, previous reports demonstrated that epithelial cells were more easily induced towards pluripotency than stromal cells. For example, it has been documented that a mesenchymal-to-epithelial transition (MET) is required to induce stromal cells towards pluripotency, and that prevention of epithelial-to mesenchymal transition (EMT) can produce iPSCs without introducing *KLF4* and *c-MYC* in epithelial cells culture[27].

Recently, it has been reported that mouse conjunctiva can be reprogrammed into iPSCs and may provide an alternative source of stem cells[28]. Our data indicates that human ocular epithelial cells (OECs) can be induced to pluripotency with a higher efficiency than conjunctival stromal cells (OSCs). Indeed, SOX2 is expressed in many epithelial cell types including conjunctival epithelial cells. More importantly, we found that in conjunctival ocular surface epithelial cells (OECs), but not ocular stromal cells (OSCs), the crucial pluripotent stem cell marker, OCT4A was expressed. The presence of these pluripotency markers in OECs may be associated with an advanced status for cell reprogramming. Previous reports indicated that OCT4 alone is sufficient to reprogram adult mouse and human neural stem cells to iPSCs because endogenous SOX2 is expressed by neural stem cells [29, 30]. Nonetheless, we could not omit *OCT4* to induce OECs to pluripotency with classic Yamanaka’s 4 factors. In fact, the OCT 4 protein level was much lower in OECs than in OECiPSCs. Whether introduction of OCT4 factor alone can induce OECs to iPSCs status requires further investigations on this traditional KMOS method. It has also been revealed that reprogramming mouse fibroblast cells into pluripotent cells can be achieved by small-molecule compounds [31]. A more recent study demonstrated that mouse somatic cells could be reprogrammed by depleting Mbd3 together with KMOS transduction on a efficiency of ~100% [32].

Treatment with such compounds or Mbd3 suppression on human fibroblast cells has not been employed in human iPSCs generation. Since activation of OCT4 to appropriately high level is required for chemical-induced pluripotency reprogramming, it would be of great interest to test such compounds on human OECs instead of fibroblast cells for chemically induced pluripotency reprogramming.

During stem cells/progenitor cells differentiation, *PAX6* is responsible for ocular development and normal limbal stem cell activity. *PAX6* knockout mice have no lenses[33]. In our microarray and RT real-time PCR data, compared with stromal cell-derived iPSCs (IMR90iPSCs and OSCiPSCs), the *PAX6* expression level in OECiPSCs was higher which may be associated with great potentials of OECiPSCs to differentiate into ocular epithelial lineage. Nonetheless, it is possible that these OECiPSCs or OSCiPSCs are induced at pluripotent state but not reporgrammed to the ground state of pluripotency, even these OECiPSCs formed teratoma containing cell types from three germ layers.

Taken together, our findings demonstrate that conjunctival epithelial cells (OECs) can be more easily converted to iPSCs than stromal cells. This cell type may also have advantages in retinal pigmented epithelial differentiation.

## Supporting Information

S1 Fig General information of ocular samples and iPSCs generation. Ocular samples 1–3 were collected and each conjunctiva tissue was digested with Dispase II overnight, and separated under stereo microscope into two layers, the OECs layer and OSCs layer. The separated layers were cultured in different conditioned medium for OSCs and OECs outgrowth. The established OEC1, OSC1, OEC2, OSC2, OEC3 and OSC3 lines were characterized by RT-PCR and immunofluorescence for their respective ocular identity. They were subjected to pluripotency reprogramming for iPSCs and their respective reprogramming efficiencies were calculated by AP-staining. The corresponding iPSCs were characterized and differentiated to ocular epithelial cell type under standard protocols provided in materials and methods.
(PDF)

S2 Fig A summary of antibodies used in the study. **(1)** Antibodies for the characterization of iPSCs with OCT4A, SOX2, NANOG, SSEA4 and TRA-1-81, **(2)** Antibodies for the characterization of iPSCs in ocular differentiation with K19, K3, P63 and RPE65. **(3)** Antibodies for identifying OCT4A and SOX2 expression in Western blotting analysis.
(PDF)

S3 Fig Primer sequences of pluripotency genes for RT-PCR in this study. To test the expression of pluripotency genes in ESCs, OECs and OSCs, forward and reverse primers of the target genes were designed.
(PDF)

S4 Fig Primers sequences of selected ocular genes for microarray real time RCR validation. Primers sequences for K19, PAX6, RPE65 and GAPDH are listed.
(PDF)

S5 Fig Efficiency of retroviral supernatant infection in OSCs and OECs primary cultures. Cells were infected with same viral supernatant harvested from PMX-GFP (retroviral) vector-transfected 293 cell cultures. The cells were subjected to two rounds of infection within 48-hours. Both of OSCs and OECs were highly infected with retroviral particles (GFP-positive) at similar percentages and fluorescent intensities **(i-ii)** OECs and **(iii-iv)** OSCs.
(PDF)

S6 Fig Methylation Analysis of *OCT4* Promoter. The biotin labeled amplification primers and the pyrosequencing primers of human *OCT 4* promoter.
(PDF)

S7 Fig Bisulfite converted amplicons of human *OCT4* promoter. Unmethylated Cytosines (C) were converted to Uracil (U) and then to Thymine (T) which were typed in red. Cytosines (methylated) on predicted CpG Islands were replaced with ‘Y’ highlighted with purple. The sequences of the pyrosequencing primers are underlined. Sequences highlighted in yellow were pyrosequencing covered regions.
(PDF)

S8 Fig Microarray data on the top 20 up-regulated genes in OEC2 compared with OSC. The genes were ranked in descending order by their corresponding mean fold changes (normalized microarray signal) for OEC2 vs OSC. NIH DAVID Pathway Analysis was used to classify the biological functions for each gene up-regulated in OEC2.
(PDF)

S9 Fig Immunostaining against K19, P63 and RPE65 markers in OECiPSCs-induced teratoma sections. **(i)** Abundant K19-positive cells; **(ii)** P63-positive cells (corneal progenitor marker) and **(iii)** RPE65-positive cells (Retinal pigmented epithelial marker) were detected. (i) Many K19-positive cells were preferentially distributed at inner layer of lumen tissues; (ii) P63- positive cells were generally distributed in the tissue, (iii) RPE65-positive cells were enriched regionally forming clustered areas within the tissue.
(PDF)

S10 Fig Microarray analysis of some important ocular genes up-regulated in OECiPSCs when compared with ESCs and OSCiPSCs. **(1)** Gene expression for COL3A1, PAX6 and SOX2 of OECiPSCs compared with ESCs; **(2)** Gene expression of COL3A1, PAX6, RPE65 and SOX2 of OECiPSCs are compared with OSCiPSCs.
(PDF)

## References

[1] Takahashi K, Tanabe K, Ohnuki M, Narita M, Ichisaka T, Tomoda K, et al Induction of pluripotent stem cells from adult human fibroblasts by defined factors. Cell. 2007;131(5):861–72. Epub 2007/11/24. 10.1016/j.cell.2007.11.019 . 18035408

[2] Tost J, Gut IG. DNA methylation analysis by pyrosequencing. Nature protocols. 2007;2(9):2265–75. Epub 2007/09/15. 10.1038/nprot.2007.314 . 17853883

[3] Zanudin A, Burns Y, Gray PH, Danks M, Poulsen L, Watter P. Perinatal Events and Motor Performance of Children Born With ELBW and Nondisabled. Pediatr Phys Ther. 2013;25(1):30–5. Epub 2013/01/05. 10.1097/PEP.0b013e31827aa424 . 23288005

[4] Rasmussen MA, Holst B, Tumer Z, Johnsen MG, Zhou S, Stummann TC, et al Transient p53 suppression increases reprogramming of human fibroblasts without affecting apoptosis and DNA damage. Stem cell reports. 2014;3(3):404–13. Epub 2014/09/23. 10.1016/j.stemcr.2014.07.006 25241739 PMC4266010

[5] Liu SP, Li YX, Xu J, Gu HH, Zhang HY, Liang HY, et al [An improved method for generating integration-free human induced pluripotent stem cells]. Zhongguo shi yan xue ye xue za zhi / Zhongguo bing li sheng li xue hui = Journal of experimental hematology / Chinese Association of Pathophysiology. 2014;22(3):580–7. Epub 2014/07/06. 10.7534/j.issn.1009-2137.2014.03.002 . 24989258

[6] Montserrat N, Nivet E, Sancho-Martinez I, Hishida T, Kumar S, Miquel L, et al Reprogramming of human fibroblasts to pluripotency with lineage specifiers. Cell Stem Cell. 2013;13(3):341–50. Epub 2013/07/23. 10.1016/j.stem.2013.06.019 . 23871606

[7] Shu J, Wu C, Wu Y, Li Z, Shao S, Zhao W, et al Induction of pluripotency in mouse somatic cells with lineage specifiers. Cell. 2013;153(5):963–75. Epub 2013/05/28. 10.1016/j.cell.2013.05.001 . 23706735 PMC4640445

[8] Yu J, Vodyanik MA, Smuga-Otto K, Antosiewicz-Bourget J, Frane JL, Tian S, et al Induced pluripotent stem cell lines derived from human somatic cells. Science. 2007;318(5858):1917–20. Epub 2007/11/22. 10.1126/science.1151526 . 18029452

[9] Park IH, Arora N, Huo H, Maherali N, Ahfeldt T, Shimamura A, et al Disease-specific induced pluripotent stem cells. Cell. 2008;134(5):877–86. Epub 2008/08/12. 10.1016/j.cell.2008.07.041 18691744 PMC2633781

[10] Aasen T, Raya A, Barrero MJ, Garreta E, Consiglio A, Gonzalez F, et al Efficient and rapid generation of induced pluripotent stem cells from human keratinocytes. Nat Biotechnol. 2008;26(11):1276–84. Epub 2008/10/22. 10.1038/nbt.1503 . 18931654

[11] Giorgetti A, Montserrat N, Aasen T, Gonzalez F, Rodriguez-Piza I, Vassena R, et al Generation of induced pluripotent stem cells from human cord blood using OCT4 and SOX2. Cell Stem Cell. 2009;5(4):353–7. Epub 2009/10/03. 10.1016/j.stem.2009.09.008 19796614 PMC2779776

[12] Haase A, Olmer R, Schwanke K, Wunderlich S, Merkert S, Hess C, et al Generation of induced pluripotent stem cells from human cord blood. Cell Stem Cell. 2009;5(4):434–41. Epub 2009/10/03. 10.1016/j.stem.2009.08.021 . 19796623

[13] Esteban MA, Wang T, Qin B, Yang J, Qin D, Cai J, et al Vitamin C enhances the generation of mouse and human induced pluripotent stem cells. Cell Stem Cell. 2010;6(1):71–9. Epub 2009/12/29. 10.1016/j.stem.2009.12.001 . 20036631

[14] Cai J, Li W, Su H, Qin D, Yang J, Zhu F, et al Generation of human induced pluripotent stem cells from umbilical cord matrix and amniotic membrane mesenchymal cells. J Biol Chem. 2010;285(15):11227–34. Epub 2010/02/09. 10.1074/jbc.M109.086389 20139068 PMC2857000

[15] Zhou T, Benda C, Duzinger S, Huang Y, Li X, Li Y, et al Generation of induced pluripotent stem cells from urine. J Am Soc Nephrol. 2011;22(7):1221–8. Epub 2011/06/04. 10.1681/ASN.2011010106 21636641 PMC3137570

[16] Lian Q, Zhang Y, Zhang J, Zhang HK, Wu X, Lam FF, et al Functional mesenchymal stem cells derived from human induced pluripotent stem cells attenuate limb ischemia in mice. Circulation. 2010;121(9):1113–23. Epub 2010/02/24. 10.1161/CIRCULATIONAHA.109.898312 . 20176987

[17] Warthemann R, Eildermann K, Debowski K, Behr R. False-positive antibody signals for the pluripotency factor OCT4A (POU5F1) in testis-derived cells may lead to erroneous data and misinterpretations. Molecular human reproduction. 2012;18(12):605–12. Epub 2012/08/31. 10.1093/molehr/gas032 22933709 PMC3497886

[18] Zhou SY, Zhang C, Baradaran E, Chuck RS. Human corneal basal epithelial cells express an embryonic stem cell marker OCT4. Current eye research. 2010;35(11):978–85. Epub 2010/10/21. 10.3109/02713683.2010.516465 . 20958186

[19] Chen SY, Hayashida Y, Chen MY, Xie HT, Tseng SC. A new isolation method of human limbal progenitor cells by maintaining close association with their niche cells. Tissue engineering Part C, Methods. 2011;17(5):537–48. Epub 2010/12/24. 10.1089/ten.TEC.2010.0609 21175372 PMC3129703

[20] Harun MH, Sepian SN, Chua KH, Ropilah AR, Abd Ghafar N, Che-Hamzah J, et al Human forniceal region is the stem cell-rich zone of the conjunctival epithelium. Human cell. 2013;26(1):35–40. Epub 2011/07/13. 10.1007/s13577-011-0025-0 . 21748521

[21] Yamanaka S. Strategies and new developments in the generation of patient-specific pluripotent stem cells. Cell Stem Cell. 2007;1(1):39–49. Epub 2008/03/29. 10.1016/j.stem.2007.05.012 . 18371333

[22] Takahashi K, Yamanaka S. Induction of pluripotent stem cells from mouse embryonic and adult fibroblast cultures by defined factors. Cell. 2006;126(4):663–76. Epub 2006/08/15. 10.1016/j.cell.2006.07.024 . 16904174

[23] Wagner JR, Busche S, Ge B, Kwan T, Pastinen T, Blanchette M. The relationship between DNA methylation, genetic and expression inter-individual variation in untransformed human fibroblasts. Genome Biol. 2014;15(2). doi: Artn R37 10.1186/Gb-2014-15-2-R37 . PMC4053980 24555846

[24] Papini S, Rosellini A, Nardi M, Giannarini C, Revoltella RP. Selective growth and expansion of human corneal epithelial basal stem cells in a three-dimensional-organ culture. Differentiation. 2005;73(2–3):61–8. Epub 2005/04/07. 10.1111/j.1432-0436.2005.07302006.x . 15811129

[25] Kim K, Doi A, Wen B, Ng K, Zhao R, Cahan P, et al Epigenetic memory in induced pluripotent stem cells. Nature. 2010;467(7313):285–90. Epub 2010/07/21. 10.1038/nature09342 20644535 PMC3150836

[26] Loh YH, Hartung O, Li H, Guo C, Sahalie JM, Manos PD, et al Reprogramming of T cells from human peripheral blood. Cell stem cell. 2010;7(1):15–9. Epub 2010/07/14. 10.1016/j.stem.2010.06.004 20621044 PMC2913590

[27] Li R, Liang J, Ni S, Zhou T, Qing X, Li H, et al A mesenchymal-to-epithelial transition initiates and is required for the nuclear reprogramming of mouse fibroblasts. Cell Stem Cell. 2010;7(1):51–63. Epub 2010/07/14. 10.1016/j.stem.2010.04.014 . 20621050

[28] Yang J, Li Y, Erol D, Wu WH, Tsai YT, Li XR, et al Generation of induced pluripotent stem cells from conjunctiva. Graefes Arch Clin Exp Ophthalmol. 2014;252(3):423–31. Epub 2014/02/05. 10.1007/s00417-014-2575-9 . 24492934 PMC3974167

[29] Loh YH, Agarwal S, Park IH, Urbach A, Huo H, Heffner GC, et al Generation of induced pluripotent stem cells from human blood. Blood. 2009;113(22):5476–9. Epub 2009/03/21. 10.1182/blood-2009-02-204800 19299331 PMC2689048

[30] Kim D, Kim CH, Moon JI, Chung YG, Chang MY, Han BS, et al Generation of human induced pluripotent stem cells by direct delivery of reprogramming proteins. Cell stem cell. 2009;4(6):472–6. Epub 2009/06/02. 10.1016/j.stem.2009.05.005 19481515 PMC2705327

[31] Hou P, Li Y, Zhang X, Liu C, Guan J, Li H, et al Pluripotent stem cells induced from mouse somatic cells by small-molecule compounds. Science. 2013;341(6146):651–4. Epub 2013/07/23. 10.1126/science.1239278 . 23868920

[32] Rais Y, Zviran A, Geula S, Gafni O, Chomsky E, Viukov S, et al Deterministic direct reprogramming of somatic cells to pluripotency. Nature. 2013;502(7469):65–70. Epub 2013/09/21. 10.1038/nature12587 . 24048479

[33] Grindley JC, Davidson DR, Hill RE. The role of Pax-6 in eye and nasal development. Development. 1995;121(5):1433–42. Epub 1995/05/01. . 7789273 10.1242/dev.121.5.1433

