# Supplementary material for: Human Ocular Epithelial Cells Endogenously Expressing SOX2 and OCT4 Yield High Efficiency of Pluripotency Reprogramming
Source: PLoS One. 2015 Jul 1;10(7):e0131288. doi: 10.1371/journal.pone.0131288 (PMC4489496; doi:10.1371/journal.pone.0131288)
Supplement: S1 Fig — Ocular samples 1–3 were collected and each conjunctiva tissue was digested with Dispase II overnight, and separated under stereo microscope into two layers, the OECs layer and OSCs layer. The separated layers were cultured in different conditioned medium for OSCs and OECs outgrowth. The established OEC1, OSC1, OEC2, OSC2, OEC3 and OSC3 lines were characterized by RT-PCR and immunofluorescence for their respective ocular identity. They were subjected to pluripotency reprogramming for iPSCs and their respective reprogramming efficiencies were calculated by AP-staining. The corresponding iPSCs were characterized and differentiated to ocular epithelial cell type under standard protocols provided in materials and methods. (PDF) [file pone.0131288.s001.pdf]

Supplementary Figure S1

General Information of Conjunctival Samples and iPSCs Generation

Ocular Samples		Conjunctival Sample 1		Conjunctival Sample 2		Conjunctival Sample 3	
Gender & ethnic		M		F		M	
		Asian		Asian		Asian	
Cell type		OEC1	OSC1	OEC2	OSC2	OEC3	OSC3
Donor cell characterization	qPCR	√	√	√	√	√	√
	IF	√	√	√	√	√	√
Passage of Cells for reprogramming		P2	P2	P2	P2	P2	N/A
Reprogramming efficiency		1.50%	0.10%	1.60%	0.08%	2.00%	N/A
Number of iPSC colonies picked		4	5	4	4	5	N/A
iPSC characterization	qPCR	√	√	√	√	√	N/A
	IF	√	√	√	√	√	N/A
	DNA methylation	√	√	N/A	N/A	N/A	N/A
	Karyotype	√	√	√	√	√	N/A
	DNA microarrays	√	√	√	N/A	N/A	N/A
	Teratoma	√	√	√	√	√	N/A
Used for differentiation	Epithelial differentiation	√	√	√	√	√	N/A
	Retinal epithelial differentiation	√	√	√	√	√	N/A
