# Supplementary material for: Human Ocular Epithelial Cells Endogenously Expressing SOX2 and OCT4 Yield High Efficiency of Pluripotency Reprogramming
Source: PLoS One. 2015 Jul 1;10(7):e0131288. doi: 10.1371/journal.pone.0131288 (PMC4489496; doi:10.1371/journal.pone.0131288)
Supplement: S2 Fig — (1) Antibodies for the characterization of iPSCs with OCT4A, SOX2, NANOG, SSEA4 and TRA-1-81, (2) Antibodies for the characterization of iPSCs in ocular differentiation with K19, K3, P63 and RPE65. (3) Antibodies for identifying OCT4A and SOX2 expression in Western blotting analysis. (PDF) [file pone.0131288.s002.pdf]

Supplementary Figure S2

A Summary of Antibodies Used in the Study

Markers	Cat. No.	Company
**Markers for pluriopentcy**		
OCT4A	SC-365509	Santa Cruz
SOX2	SC-17320	Santa Cruz
NANOG	SC-33759	Santa Cruz
SSEA4	90231	Millipore
TRA-1-81	SC 21706	Santa Cruz
**Markers for characterization of iPSCs differeniated ocular cells**		
K19	SC-56371	Santa Cruz
K3	SC-49179	Santa Cruz
P63	SC-8431	Santa Cruz
RPE65	SC-32893	Santa Cruz
**Antibodies for Western Blotting**		
OCT4A	SC-365509	Santa Cruz
SOX2	SC-17320	Santa Cruz
Beta Actin	SC-47778	Santa Cruz
