# Supplementary material for: Human Ocular Epithelial Cells Endogenously Expressing SOX2 and OCT4 Yield High Efficiency of Pluripotency Reprogramming
Source: PLoS One. 2015 Jul 1;10(7):e0131288. doi: 10.1371/journal.pone.0131288 (PMC4489496; doi:10.1371/journal.pone.0131288)
Supplement: S3 Fig — To test the expression of pluripotency genes in ESCs, OECs and OSCs, forward and reverse primers of the target genes were designed. (PDF) [file pone.0131288.s003.pdf]

Supplementary Figure S3

Primer Sequences of Pluripotency Genes for RT-PCR

Gene	Primers	Accession
h-endo OCT4A	CTCTGAGGTGTGGGGGATT<br>TGCTCCAGCTTCTCCTTCTC	NM_002701.3
h-endo SOX2	GGGAAATGGGAGGGGTGCAAAAGAGG<br>TTGCGTGAGTGTGGATGGGAT TGGTG	NM_003106.3
h-endo c-MYC	GCGTCCTGGGAAGGGAGATCCGGAGC<br>TTGAGGGGCATCGTCGCGGGAGGCTG	NM_002467.4
h-endo KLF4	ACGATCGTGGCCCCGGAAAAGGACC<br>TGATTGTAGTGCTTTCTGGCTGGGCTCC	NM_004235.4
h-NANOG	CAGCCCCGATTCTTCCACCAGTCCC<br>CGGAAGATTCCCAGTCGGGTTCACC	NM_024865.2
h-GDF3	CTTATGCTACGTAAAGGAGCTGGG<br>GTGCCAACCCAGGTCCCGGAAGTT	NM_020634.1
h-REX1	CAGATCCTAAACAGCTCGCAGAAT<br>GCGTACGCAAATTAAAGTCCAGA	NM_174900.3
h-ESG1	ATATCCCGCCGTGGGTGAAAGTTC<br>ACTCAGCCATGGACTGGAGCATCC	NM_001025290.2
h-DPPA4	GGAGCCGCCTGCCCTGGAAAATTC<br>TTTTTCCTGATATTCTATTCCCAT	NM_018189.3
h-TERT	CCTGCTCAAGCTGACTCGACACCGTG<br>GGAAAAGCTGGCCCTGGGGTGGAGC	NM_001193376.1
h- β -actin	TGACGTGGACATCCGCAAAG<br>CTGGAAGGTGGACAGCGAGG	NM_001101.3

Primer Sequences for Checking the Expression of OCT4 Isoforms, Edogenous and Virual OCT4 Expression on RT-PCR

Genes	Primers
EndoOct4a	CTTCGCAAGCCCTCATTTCA
	GCCCATCACCTCCACCACC
EndoOct4b	GACATTTGTGGGTAGGTTATTTC
	CCCTTTCCATTCGGGATTCA
Viral OCT4	CCCTGTCTCTGTCACCACTCTG
	AACTGGTAATGGTAGCGACCG
GAPDH	AAGGTGAAGGTCGGAGTCA
	AATGAAGGGGTCATTGATG
