# Supplementary material for: Human Ocular Epithelial Cells Endogenously Expressing SOX2 and OCT4 Yield High Efficiency of Pluripotency Reprogramming
Source: PLoS One. 2015 Jul 1;10(7):e0131288. doi: 10.1371/journal.pone.0131288 (PMC4489496; doi:10.1371/journal.pone.0131288)
Supplement: S4 Fig — Primers sequences for K19, PAX6, RPE65 and GAPDH are listed. (PDF) [file pone.0131288.s004.pdf]

Supplementary Figure S4

Primer Sequences of Selected Ocular Genes for Microarray Real-Time PCR Validation

IDT primer Name	Accession number	Foward primer	Reverse primer
h_KRT19	NM_002276	AAGGCCTGAAGGAAGAGCTG	GGAATCCACCTCCACACTGA
h_PAX6	NM_000280.3	CGGCAGAAGATTGTAGAGC	GGATTTCCCAAGCAAAGAT
h_RPE65	NM_000329.2	CGTATGGACTTGGCTTGAATC	CTGGGTGAGAAACAAAGATGG
h_GAPDH	NM_002046	TCCCTGAGCTGAACGGGAAG	GGAGGAGTGGGTGTCGCTGT
