# Supplementary figures and images for: Human Ocular Epithelial Cells Endogenously Expressing SOX2 and OCT4 Yield High Efficiency of Pluripotency Reprogramming
Source: PLoS One. 2015 Jul 1;10(7):e0131288. doi: 10.1371/journal.pone.0131288 (PMC4489496; doi:10.1371/journal.pone.0131288)

## Supplementary Figure S5

### Retroviral Infection Efficiencies of OSCs and OECs

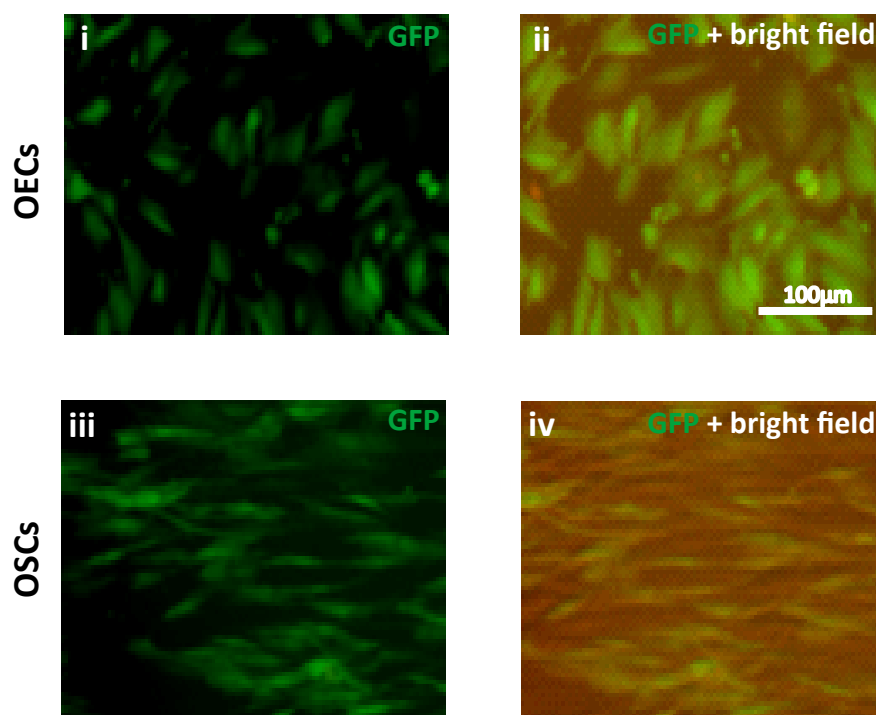

Supplement: S5 Fig — Cells were infected with same viral supernatant harvested from PMX-GFP (retroviral) vector-transfected 293 cell cultures. The cells were subjected to two rounds of infection within 48-hours. Both of OSCs and OECs were highly infected with retroviral particles (GFP-positive) at similar percentages and fluorescent intensities (i-ii) OECs and (iii-iv) OSCs. (PDF) [file pone.0131288.s005.pdf]
