# Supplementary material for: Human Ocular Epithelial Cells Endogenously Expressing SOX2 and OCT4 Yield High Efficiency of Pluripotency Reprogramming
Source: PLoS One. 2015 Jul 1;10(7):e0131288. doi: 10.1371/journal.pone.0131288 (PMC4489496; doi:10.1371/journal.pone.0131288)
Supplement: S6 Fig — The biotin labeled amplification primers and the pyrosequencing primers of human OCT 4 promoter. (PDF) [file pone.0131288.s006.pdf]

Supplementary Figure S6

Promoter Name	Primers for PCR amplification	(Biotin label)5'>3'
human *OCT4*	*Oct 4* F1	AGTTTTAGGATATTTAGGTTAGGTTTAGAA
	OCT4 R1	CAACAACCCCCCTCTACAAT
**Promoter Name**	**Primers for pyrosequencing**	
human *OCT4*	*OCT4* S1	GGATATTTAGGTTAGGTTTAGAA A
	OCT4 S2	GTATATTTTTTAATTTGTTAGGTT
