# Supplementary material for: Human Ocular Epithelial Cells Endogenously Expressing SOX2 and OCT4 Yield High Efficiency of Pluripotency Reprogramming
Source: PLoS One. 2015 Jul 1;10(7):e0131288. doi: 10.1371/journal.pone.0131288 (PMC4489496; doi:10.1371/journal.pone.0131288)
Supplement: S7 Fig — Unmethylated Cytosines (C) were converted to Uracil (U) and then to Thymine (T) which were typed in red. Cytosines (methylated) on predicted CpG Islands were replaced with ‘Y’ highlighted with purple. The sequences of the pyrosequencing primers are underlined. Sequences highlighted in yellow were pyrosequencing covered regions. (PDF) [file pone.0131288.s007.pdf]

Supplementary Figure S7

Human *OCT4* promoter

AGTTTTAGGA TATTTAGGTT AGGTTTAGAA AAATAGATTT TGAAGGGGAG
TTTAGGGTAG TTTTTTTGTA YGTTTTTATA AATTATTTTT TATTTTTTTT GYGTTTTTTT
GTTAGTTAGT TTTATTAAAT AAAGTATATT TTTTAATTTG TTAGGTTYGG GGAGGGAYGT
AYGATGAAGT TGGAYGTTTG AGTTTTTTAG AGGAAGGAGG AATTAGATAT TTAGGTTTTT
GTGGGGGGTT TTTGGTGTTY GTTTGAGGTT TAGTTTTTGAGGGGATTGTA
GAGGGGGGTT GTTG
