# Supplementary material for: Human Ocular Epithelial Cells Endogenously Expressing SOX2 and OCT4 Yield High Efficiency of Pluripotency Reprogramming
Source: PLoS One. 2015 Jul 1;10(7):e0131288. doi: 10.1371/journal.pone.0131288 (PMC4489496; doi:10.1371/journal.pone.0131288)
Supplement: S8 Fig — The genes were ranked in descending order by their corresponding mean fold changes (normalized microarray signal) for OEC2 vs OSC. NIH DAVID Pathway Analysis was used to classify the biological functions for each gene up-regulated in OEC2. (PDF) [file pone.0131288.s008.pdf]

Supplementary Figure S8

The Top 20 Genes Preferentially Up-regulated in OEC2

Table X Top 20 up-regulated genes in OEC compared with OSC

Genes	Array identifier	Fold change	Involved Biological process description
AREGB; AREG	205239_at	601.7	EGF-like
Sprr1b	205064_at	533.85	cytoskeleton, non-membrane-bounded organelle
S100A14	218677_at	458.98	metal ion binding, cation binding, ion binding
znf750	219995_s_at	413.1	cation binding, ion binding
CLCA2	206164_at	325.11	transmembrane region, integral to membrane, ion binding
SFN	33323_r_at	236.07	regulation of cell proliferation, apoptosis, cell death
Krt14	209351_at	199.51	cytoskeleton, non-membrane-bounded organelle
CLCA2	206165_s_at	180.19	transmembrane region, integral to membrane, ion binding
SORL1	212560_at	154.8	EGF-like, transmembrane region, integral to membrane
FAM83A	238460_at	147.42	
TNS4	230398_at	144.26	apoptosis, cell death, cytoskeleton, non-membrane-bounded organelle
TACSTD2	202286_s_at	139.47	transmembrane region, integral to membrane
TMEM40	219503_s_at	135.73	transmembrane region, integral to membrane
CD24L4; Cd24	208651_x_at	124.14	apoptosis, cell death, membrane fraction, insoluble fraction, cell fraction
CLCA2	217528_at	124.12	transmembrane region, integral to membrane, ion binding
igsf3	202421_at	118.37	transmembrane region, integral to membrane
PTGS2	1554997_a_at	116.56	EGF-like, apoptosis, cell death, membrane fraction, insoluble fraction, cell fraction, ion binding
RAB38	219412_at	114.66	membrane fraction, insoluble fraction, cell fraction
Lamb3	209270_at	110.9	
itga6	215177_s_at	102.61	transmembrane region, integral to membrane, ion binding
