# Supplementary material for: Human Ocular Epithelial Cells Endogenously Expressing SOX2 and OCT4 Yield High Efficiency of Pluripotency Reprogramming
Source: PLoS One. 2015 Jul 1;10(7):e0131288. doi: 10.1371/journal.pone.0131288 (PMC4489496; doi:10.1371/journal.pone.0131288)

# Supplementary Figure S9

The OECiPSCs-induced Teratoma Sections Stained against K19, P63 and RPE65

OECiPSCs-induced Teratoma

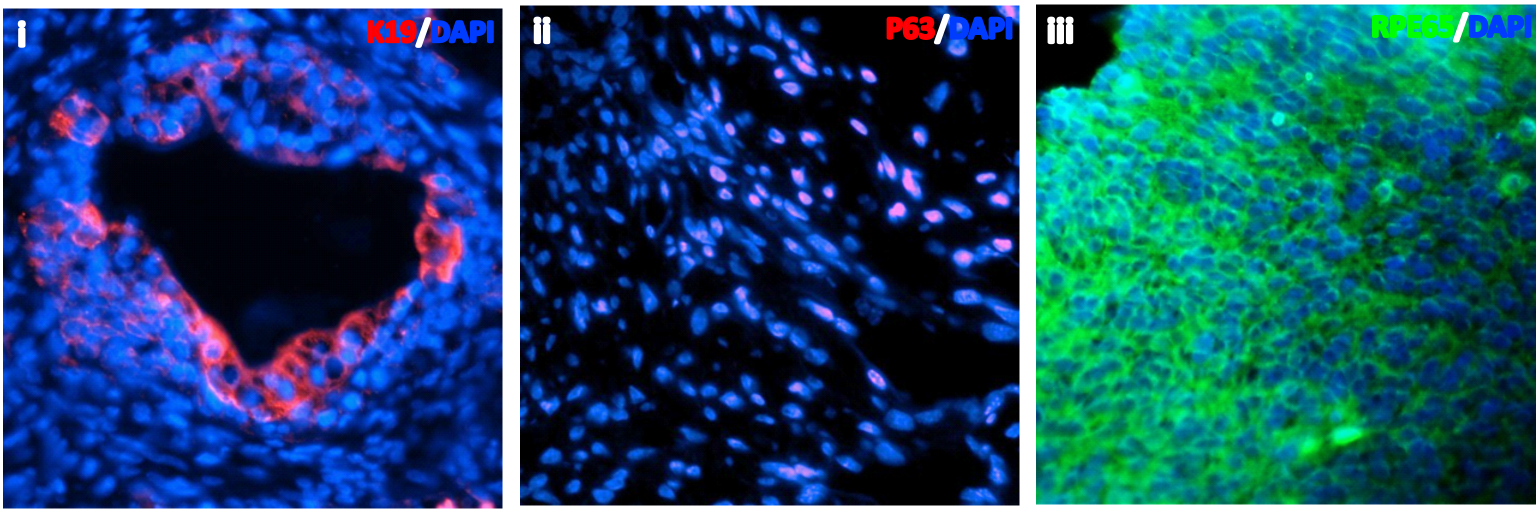

Supplement: S9 Fig — (i) Abundant K19-positive cells; (ii) P63-positive cells (corneal progenitor marker) and (iii) RPE65-positive cells (Retinal pigmented epithelial marker) were detected. (i) Many K19-positive cells were preferentially distributed at inner layer of lumen tissues; (ii) P63- positive cells were generally distributed in the tissue, (iii) RPE65-positive cells were enriched regionally forming clustered areas within the tissue. (PDF) [file pone.0131288.s009.pdf]
