# Supplementary material for: Human Ocular Epithelial Cells Endogenously Expressing SOX2 and OCT4 Yield High Efficiency of Pluripotency Reprogramming
Source: PLoS One. 2015 Jul 1;10(7):e0131288. doi: 10.1371/journal.pone.0131288 (PMC4489496; doi:10.1371/journal.pone.0131288)
Supplement: S10 Fig — (1) Gene expression for COL3A1, PAX6 and SOX2 of OECiPSCs compared with ESCs; (2) Gene expression of COL3A1, PAX6, RPE65 and SOX2 of OECiPSCs are compared with OSCiPSCs. (PDF) [file pone.0131288.s010.pdf]

Supplementary Figure S10

Important Ocular Genes Up-regulated in OECiPSCs when compared to OSCiPSCs and ESCs in Microarray Analysis

OECiPSCs vs ESCs						
Gene	Entrez Gene	Signal Intensity/ OECiPSCs	Signal Intensity/ ESCs (H9)	Up-regulated	Function in Eye Development	References
COL3A1	1281	9.61	1.12	8.58	Extracellular matrix	Prog Retin Eye Res. 2004 Jul;23(4):403-34.
PAX6	5080	3216.88	41.45	77.61	Master gene of eye development : retina and lens	Curr Opin Cell Biol. 2001 Dec;13(6):706-14.
SOX2	6657	264	63.11	4.18	Essential eye developmental components/ retinal neural progenitor	PLoS One. 2012;7(10):e47187.
**OECiPSCs vs OSCiPSCs**						
Gene	Entrez Gene	OECiPSCs	OSCiPSCs	Up-regulated	Function in Eye Development	References
COL3A1	1281	9.61	4.2	2.29	Extracellular matrix	Prog Retin Eye Res. 2004 Jul;23(4):403-34.
PAX6	5080	1284.65	159.71	8.04	Master gene of eye development : retina and lens	Curr Opin Cell Biol. 2001 Dec;13(6):706-14.
RPE65	6121	6.85	1.42	4.82	Retinal pigment epithelium protein involved in phototransduction	Prog Retin Eye Res. 2004 Jul;23(4):403-34.
SOX2	6657	264	116.32	2.27	Essential eye developmental components/ retinal neural progenitor	PLoS One. 2012;7(10):e47187.
